# Barriers of molecular epidemiology in Iran:Action plan and necessary steps

**DOI:** 10.22088/cjim.10.3.356

**Published:** 2019

**Authors:** Massoud Hajia

**Affiliations:** 1 Department of Molecular biology, Research center of Health Reference Laboratories, Ministry of Health and Medical Education, Tehran, Iran

**Dear Editor,**

Food borne pathogens are under the cover of Iranian surveillance program. According to this plan any outbreak will be investigated for the causative agents and also alerting the clinician for proper medication. However, access to the reliable data is one of earliest necessary step in any active surveillance system. New available molecular methods have now provided an excellent opportunity for us. These methods provide a better view of main pathogenic type in involved outbreaks. Undoubtedly, any plan in this regard requires close collaboration of laboratories and clinical services with Centers for Disease Control. Lack of any routinely reported reliable data on the variety of various food-borne pathogens is an important issue for the medical services (1-2). Some barriers prevent us from updating our surveillance plan, so predicting contaminated resources is not possible. 

Some of these obstacles are undoubtedly related to the required investment. But the main problem is the involved offices do not have a clear realistic vision on action plan. An integrated food-borne surveillance program certainly needs huge investment for the molecular laboratories all over the country; these labs are the main centers for investigation, isolation and identification of pathogenic strains in both human and food sources. Besides, it seems setting up a close cooperation among the involved centers under a steering center can improve current situation and ensure us for running active programs (4-6). Today, molecular epidemiology has proposed several typing systems to identify the exact source of outbreaks (7, 8). Applying reliable molecular typing techniques is important in several aspects; providing effective investigation on the main causative agent of outbreaks, giving a better view on how to expand it to the society, finding the source of contamination, and alerting for selecting proper or improved treatment protocols.

Fortunately, the necessary molecular labs, called Research Centers, exist under the Deputy of research in many universities, and there is no need for required heavy investment. Only reorganization is required (9). Another requirement is harmonizing laboratory results that need to have an accrediting body that can be a central core laboratory as well. An accrediting body can obviously support other labs` partner to provide standard operating procedures  (SOPs), checklists, and holding workshops for the training the staff. Running quality assurance program throughout all involved laboratories, and finally analyzing, approving, storing and reporting data should be under duty of a core center. But the important point currently is that it is not necessary that all pathogens be entered at the proposed plan or all the laboratories are involved. This plan can be developed step-by-step. We only need to have a well-organized system (picture 1). 

At present, pulsed-field gel electrophoresis (PFGE) is reported as the only typing method that has potential capability to be used on a surveillance program at molecular level for food borne diseases. It is very important that each lab partner participate and pass the required accrediting processes, before accepting to be a member of this network. They need to provide standard laboratory setting, having necessary instruments, and passing from accreditation program. These members must regularly take part in standard proficiency testing. Starting step should be preparing a clear proposal for this program that has been confirmed by financially supporting the authority office. The necessity of having reliable and reproducible results makes us to be ensured interferes of various parameters especially at the molecular level and even in storing images for future uses. Trustable typing patterns are quite crucial and need accurate standardization for inter-laboratory works. Therefore each laboratory must strictly follow released SOPs (10-11). Setting up standard work space to avoid contamination, staff training, monitoring reagents, appropriate control for the genome preparation and digestion are included in internal quality assurance. External quality assurance also needs to be included in the program. This program contains initially the next regular performances of the typing results. It is the required for all partners since the running protocol is going to be used in national surveillance network. Therefore, it is necessary that each lab strictly follow the SOPs. 

**Picture 1:**
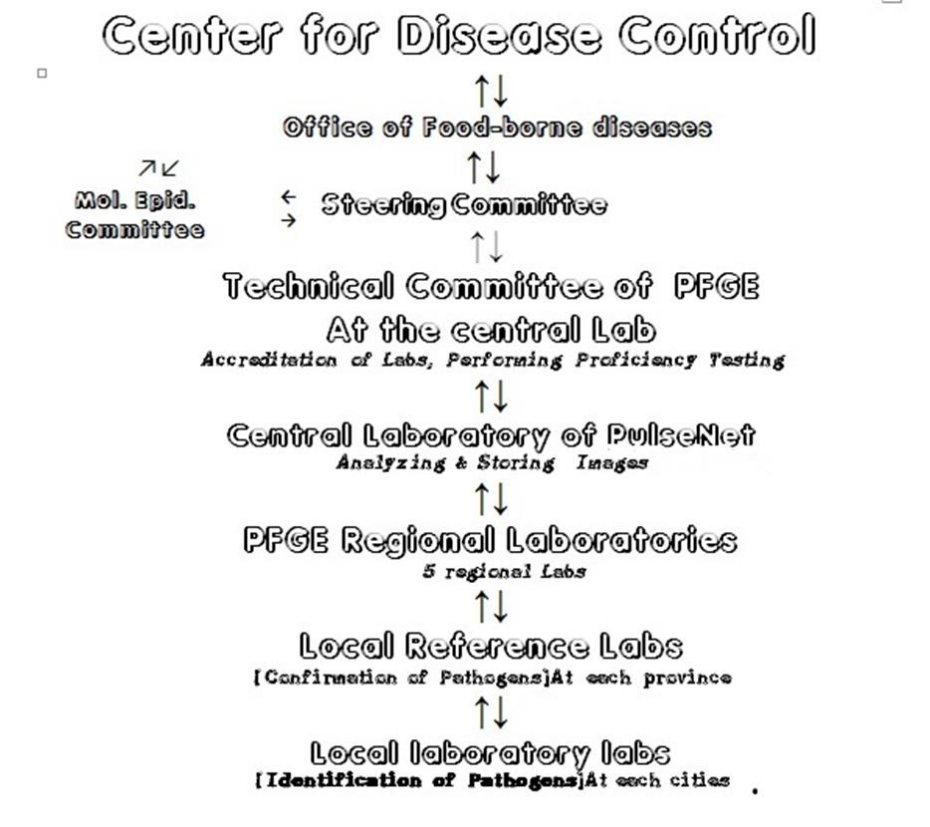
Proposed workflow for Iran

In conclusion, Setting up a new surveillance program needs to have a clear view of any possible challenges and difficulties for following-up this plan. There are several different approaches for the supervision program on food borne disease. Many countries are trying to improve their activities headed for the integrated food-borne surveillance programs. Sooner or later, we need to go toward the setting up the third system for monitoring food borne pathogens.

## References

[1] Boxrud D, Monson T, Stiles T, Besser J (2010). The role, challenges, and support of PulseNet laboratories in detecting foodborne disease outbreaks. Public Health Rep.

[2] Hajia M, Sohrabi A (2019). In Silico Characteristics for Re-emerging Possibility of Vcholerae Genotypes in Iran. New Mic New Infect.

[3] World Health Organization (2011). Activity report 2011. http://who.int/iris/bitstream/10665/70880/1/WHO_HSE_GCR_LYO_2012.3_eng.pdf?ua=1.

[4] Li W, Lu S, Cui Z (2012). PulseNet China, a model for future laboratory-based bacterial infectious disease surveillance in China. Front Med.

[5] Standard Operating Procedure for PulseNet PFGE of Vibrio cholera and Vibrio parahaemolyticus. http://www.cdc.gov/pulsenet/PDF/vibrio_pfge_protocol-508c.pdf.

[6] LiW, Wu S, Fu P (2018). National molecular tracing network for foodborne disease surveillance in China. Food Control.

[7] Hajia M, Rahbar M, Farzami MR (2015). Assessing clonal correlation of epidemic Vibrio cholerae isolates during 2011 in 16 provinces of Iran. Curr Microbiol.

[8] Hajia M, Dolatyar A, Farzami MR (2016). Evaluating correlation of the native Inaba strain with the dominant isolated strains in outbreaks occurred in Iran at 2013 by Pulsed Field Gel Electrophoresis. J Microbiol Infect Dis.

[9] Hajia M (2018). Molecular epidemiology and surveillance program in Iran; present status, and future prospect. Int J Epidemiol Res.

[10] Hajia M, Safadel N, Samiee SM (2013). Quality Assurance Program for Molecular Medicine Laboratories. Iran J Pub Health.

[11] Hajia M, Safadel N (2019). Secondary Use of Laboratory data: Potentialities and Limitations. Iranian J Path.

